# Identifying and manipulating single atoms with scanning transmission electron microscopy

**DOI:** 10.1039/d2cc04807h

**Published:** 2022-09-29

**Authors:** Toma Susi

**Affiliations:** University of Vienna, Faculty of Physics Boltzmanngasse 5 1090 Vienna Austria toma.susi@univie.ac.at

## Abstract

The manipulation of individual atoms has developed from visionary speculation into an established experimental science. Using focused electron irradiation in a scanning transmission electron microscope instead of a physical tip in a scanning probe microscope confers several benefits, including thermal stability of the manipulated structures, the ability to reach into bulk crystals, and the chemical identification of single atoms. However, energetic electron irradiation also presents unique challenges, with an inevitable possibility of irradiation damage. Understanding the underlying mechanisms will undoubtedly continue to play an important role to guide experiments. Great progress has been made in several materials including graphene, carbon nanotubes, and crystalline silicon in the eight years since the discovery of electron-beam manipulation, but the important challenges that remain will determine how far we can expect to progress in the near future.

## Abstract 2

A focused electron beam can be used to manipulate covalently bound impurities within crystal lattices with atomic precision.

## Graphical abstract

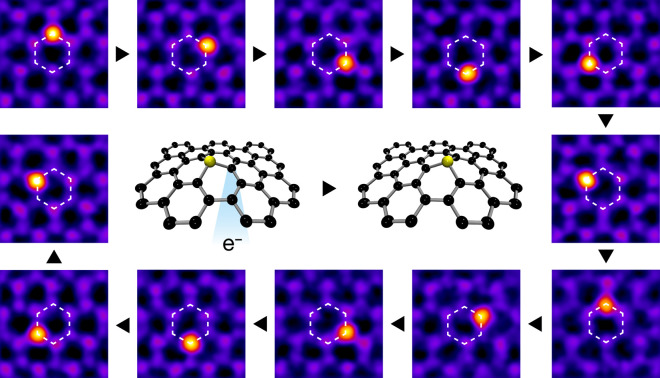

## 1 Introduction

The notion of atoms as indivisible building blocks of matter is literally ancient, reaching back to Greek philosophers Anaxagoras and Democritus. Revived by Gassendi in the late 16th century,^1^ the atomic hypothesis was expressed in its modern form by the chemist Dalton in 1803,^2^ building on the work of several others.^3^ The idea was resisted for the following century as unobservable and metaphysical,^4^ or at best as merely a convenient way of keeping account of chemical substances and their reactions.

Despite acceptance by such luminaries of mid-nineteenth century physics as Maxwell and van der Waals,^5^ towards the end of the century, the atomic hypothesis faced increasing opposition from leading scientists including Pierre Curie, Ostwald, and, most vociferously, Mach.^6^ By the start of the twentieth century, Boltzmann remained its most vigorous defender with notable support from Planck,^7^ but sadly did not live to see the final vindication of his views. These debates were finally laid to rest by Einstein's theory of Brownian motion^8^ and its experimental confirmation by Perrin in 1909.^9^

The development of modern atomic theory, prompted by the discovery of the electron by Thomson in 1897,^10^ the nucleus by Rutherford in 1911,^11^ and chemical isotopes by Soddy in 1913,^12^ revealed that although atoms are indeed indivisible in a chemical sense, they are in fact not the smallest unit of matter. Further scattering experiments and the formulation of quantum mechanics solidified our understanding of the periodic table as a sequence of atoms with an increasing number of protons, stabilized by varying numbers of neutrons in a compact nucleus, and neutralized by an equal number of oppositely charged electrons orbiting as quantized waveforms. Thus, we came to the conception of atoms as we still understand them to this day.

However, it took many decades and several scientific and technological breakthroughs before individual atoms could be directly observed and identified—let alone controllably manipulated.

## 2 Identifying single atoms

Atoms were first imaged by field ion microscopy (FIM), which was used to record atomically resolved images of a metal surface in 1955.^13^ Although FIM has been very useful for understanding phenomena including surface diffusion,^14^ it was challenging to use it for elemental identification.^15^ By augmenting this with the aid of mass spectrometry into atom probe tomography,^16^ atomically resolved information of the chemical and even isotopic structure of materials can be obtained.^17^ However, analysis entails the destruction of specially shaped samples, with no control over the real-space location of the interactions.

For the imaging and identification of single atoms in materials, including any possibility to direct their movement, two techniques stand out: scanning transmission electron microscopy and scanning probe microscopy. While the latter was both the first and, until recently, the only technique capable of atom manipulation, transmission electron microscopy (TEM) which is our focus here predates it by half a century. Let us thus start with discussing the historical development of TEM and especially its unsurpassed capabilities for identifying single atoms *via* the elastic and inelastic scattering of energetic primary beam electrons.

### 2.1 Transmission electron microscopy

Following the experiments confirming de Broglie's theory of wave-matter duality by Thomson in 1927 using electron diffraction^18^ and by Davisson and Germer in 1928 using electron reflection,^19^ it was soon realized that due to their much smaller wavelength, electron waves offered the possibility of significantly higher diffraction-limited imaging resolution than light. The first instrument operating by this principle was developed by Ruska in 1932.^20^ However, despite swift progress in the field, the first unambiguous images of single atoms were taken only in 1970 with the help of scanning transmission electron microscopy (STEM) refined by Crewe.^21^ The lower energy spread and higher brightness of a field-emission electron gun, combined with annular dark-field detection – which is sensitive to the nuclear potential – to maximize the contrast of atoms with extremely high atomic number *Z* dispersed on low-*Z* carbon support, made it possible to identify single U and Th atoms even with a 5 Å-sized electron probe. This work was later extended to lower relative *Z* differences by Treacy and Rice in 1989^22^ and to crystalline supports by Nellist and Pennycook in 1996.^23^

The first reliable identification of covalently bound impurities within a crystal lattice was reported by Voyles and colleagues in 2002,^24^ when they imaged antimony (Sb) dopant atoms in crystalline silicon – which, as we will see, is a system that has later proven fruitful for electron-beam manipulation. Using focal-series optical sectioning, even the three-dimensional location of heavier dopants can be approximately inferred from ADF images.^25,26^ However, image simulations of the Sb in silicon^24^ showed them not to be visible in phase-contrast high-resolution TEM (HRTEM), even with exit-wave reconstruction.

Thus, although the first HRTEM image resolving atomic columns is thought to have been recorded by Iijima back in 1971,^27^ the reliable identification of dopant atoms using HRTEM is much more challenging due to the complicated effects of dynamical and inelastic scattering on phase contrast. The cross-sectional images of Au and Pt atoms within graphene layers presented in 2008 by Gan^28^ are convincing, though the identity of the elements was merely inferred from sample preparation. By comparing *ab initio*-based image simulations to experimental contrast at a specific defocus value, Meyer was 2011 able to identify N substitutions in graphene by their phase contrast in HRTEM.^29^ It should also be noted that energy-filtered imaging with achromatic electron optics can directly reveal the atomic elemental structure, as demonstrated in 2013 by Urban.^30^

Phase contrast is more sensitive to low-*Z* elements than ADF, which may be unable to resolve them especially when neighboring atoms that scatter more strongly. However, modern STEM imaging techniques can largely overcome this difficulty, and light atoms including H can be resolved with annular bright-field (ABF) detection^31^ or integrated differential phase contrast (iDPC).^32,33^ Recently, it was also somewhat surprisingly demonstrated that secondary electron signals can also be collected from single atoms.^34^ This is due to a substantial part arising from inner-shell excitations that are more localized than valence excitations even at primary beam energies used in scanning electron microscopy.^35^ However, there is little elemental sensitivity, and so without collecting an X-ray spectrum, this technique cannot be used to reliably identify atomic species.

The development of effective computer-controlled aberration correctors over the past three decades^36^ has made it possible to focus STEM electron beams to sub-Ångström spots^37^ and to correct image aberrations in HRTEM to reach similar resolutions.^38^ While the improved spatial resolution has made it possible to convincingly distinguish light elements^39^ with realistic ADF noise, it is hard to conclusive discriminate between similar heavier elements such as aluminum, silicon and phosphorus.^40^ Thus, it was the combination of atomically focused electron beams with efficient spectrometers in aberration-corrected STEMs that has been the crucial breakthrough in unambiguously identifying single atoms.

### 2.2 Atomically resolved spectroscopy

Primarily two types of spectroscopic signatures can be used for elemental identification, namely characteristic X-rays generated by the radiative recombination of inner-shell excitations (energy-dispersive X-ray, EDX) and the core energy-loss of primary beam electrons (electron energy-loss spectroscopy, EELS). Due to the dependence of the probability of radiative recombination on the atomic number *Z*, EDX is typically measurable only for heavier elements, whereas EELS is stronger for lighter ones. For our purposes, it is of interest to divide pioneering studies into two categories: atomically resolved spectroscopy, where distinct spectral signals can be shown to arise from neighboring atoms or atomic columns, and single-atom spectroscopy, where the signature of only a single impurity atom is detected. Three preconditions need to be fulfilled:^41^ sufficient resolution with respect to the separation of atoms; sufficient signal to noise with respect to the background and signatures of other nearby elements; and sufficient sample and instrument stability to record the signal.

Atomically resolved EELS was reported by two groups in 1993: Batson observed the different bonding states of silicon atoms across a Si–SiO_2_ interface,^42^ and Browning studied the epitaxial interfaces between cobalt silicide and silicon.^43^ Spatial resolution in such studies is limited not only by the size of the electron probe and possible beam spreading in the specimen but ultimately by the delocalization of the studied excitations.^44^ Due to instrumental improvements that allowed two-dimensional EELS maps to be practically acquired,^45,46^ it is possible to localize dopants in bulk crystals using EELS even if their scattering contrast is too weak to directly detect in images,^47,48^ but the speed of such measurements is limited. Atomically resolved EDX maps were first reported by D'Alfonso in 2010.^49^

**Fig. 1:**
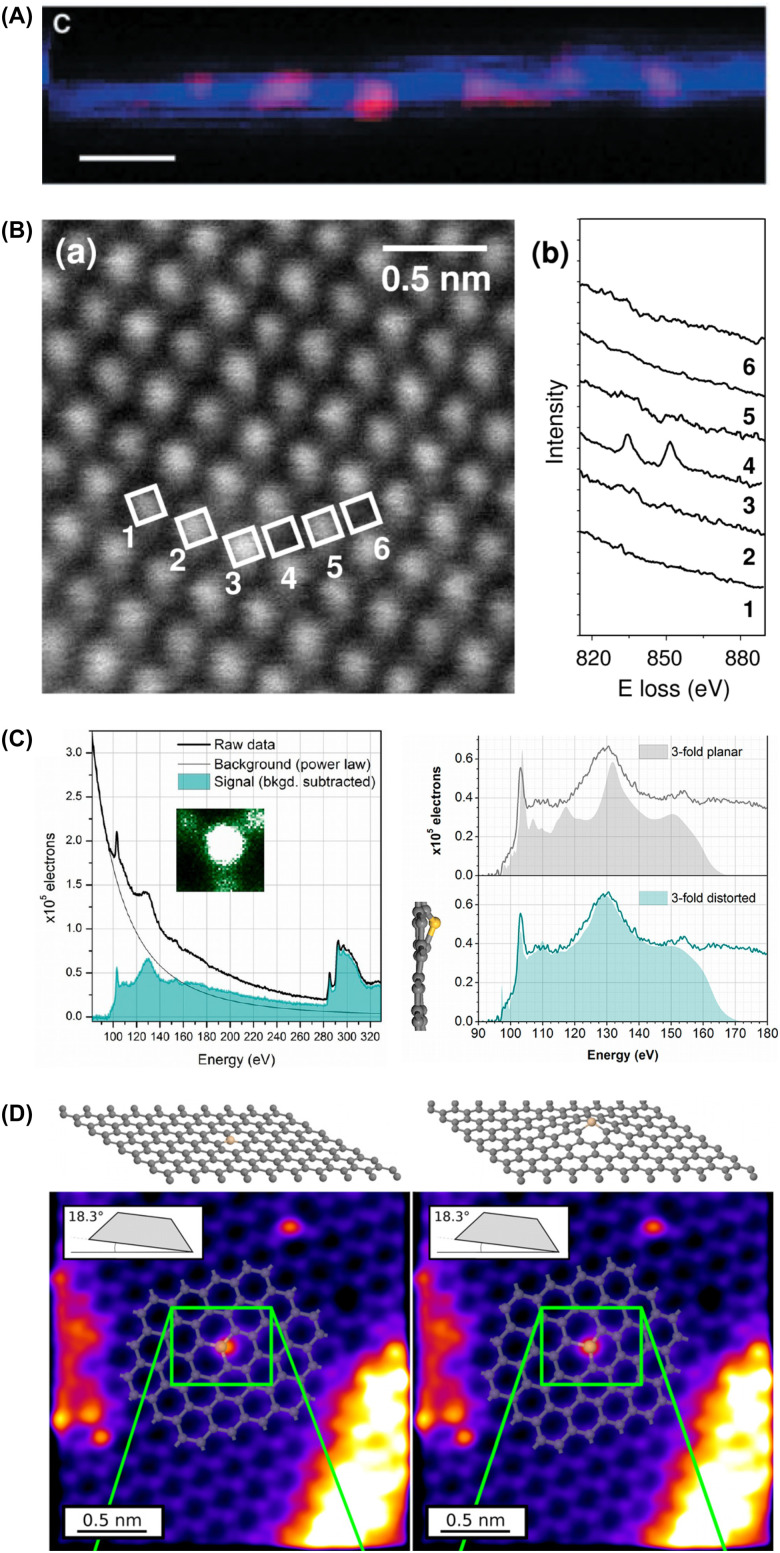
Identifying single atoms with scanning transmission electron microscopy and spectroscopy. (A) EEL spectrum maps of gadolinium atoms (Gd *N* edge signal, red) encapsulated in fullerenes filling a single-walled carbon nanotube (C *K* edge, blue). The scale bar is 3 nm. From ref. 50. Reprinted with permission from AAAS. (B) Annular dark-field STEM image of CoTiO_3_, with atomic-column EEL spectra showing a La *M*_4,5_ edge signature on site 4 (numbered white square). Adapted with permission from ref. 51. Copyright (2004) by the American Physical Society. (C) Core-loss EEL signal integrated over a three-coordinated silicon impurity in graphene (inset shows the corresponding ADF signal), and spectrum simulations for a flat and out-of-plane distorted structure compared to the Si *L*_2,3_ edge signal. Adapted with permission from ref. 52. Copyright 2013 American Chemical Society. (D) Two-tilt tomographic reconstruction of the out-of-plane distortion of the Si site. Adapted from ref. 53, with the permission of AIP Publishing.

The first single-atom EELS studies were published by Suenaga in 2000^50^ on gadolinium atoms confined within fullerenes filling single-walled carbon nanotubes (Fig. 1), and EEL spectra of single covalently bound lanthanum lattice impurities in CoTiO_3_ were recorded by Varela in 2004.^51^ More recent work has shown that single-atom sensitivity can be retained even for vibrational excitations in suitable samples.^54^ Single-atom X-ray analysis was first reported in 2012 for erbium inside fullerene cages by Suenaga^55^ and for silicon and platinum substitutions in graphene by Lovejoy.^56^ Analysis of the EEL fine structure has even allowed the spin states of individual atoms to be determined,^57,58^ and light elements down to lithium be identified.^59^

Covalently bound lattice impurities in metallic – and thus radiation-resistant – one-atom-thick graphene provide arguably the ideal system for single-atom studies. Silicon impurities were measured by Chisholm and by Zhou in 2012,^60,61^ soon followed by N and B by Bangert.^62^ A detailed comparison of first-principles EEL spectrum simulations with single-atom experimental spectra makes it possible to infer even the three-dimensional structure of the impurity site,^52,63^ which can be verified using tilting experiments.^53^ Thus, single lattice impurities in graphene are some of the most carefully characterized systems, and due to their importance for the discovery of atom manipulation using STEM, the available literature is summarized in Table 1.

**Table 1:** Identification of individual impurity atoms in graphene with transmission electron microscopy, and the type of imaging and spectroscopic evidence presented in each case. “Indirect”' indicates that spectra were collected elsewhere on the sample

Element(s)	Contrast	Spectroscopy	Ref.
Pt, Au	Phase	—	28
Pt, Co, In	Phase	—	64
Si, Pt	ADF	Single-atom EDX	56
Si, Fe	ADF	Single-atom EELS	60
Fe	Phase	—	65 and 66
Si	ADF	Single-atom EELS	52, 61 and 67
N & B	ADF	Single-atom EELS	62
N	ADF	Single-atom EELS	68
N, Fe, Cr	ADF	Single-atom EELS	57
N, Fe	ADF	Single-atom EELS	69
Ni	ADF	Indirect EDX	70
P	ADF	Single-atom EELS	63, 71 and 72
Ge	ADF	Indirect EDX	73
O	ADF	Single-atom EELS	53
Si, Pt	ADF	Indirect EELS	74
Several metals	ADF	Indirect EELS	75
Mn	ADF	Single-atom EELS	58
Au	ADF	Single-atom EELS	76
Al	phase	Areal EELS	77
Al	ADF	Single-atom EELS	40

However, when direct and atomically resolved spectroscopic evidence is not available, caution must be taken in assigning the identity of elements observed in images, and especially when phase contrast is involved even in atomically thin material such as graphene. Numerous studies have assigned elemental identities to individual atoms based on their contrast, but extreme sensitivity to imaging conditions compounded by the lack of an atomically localized spectroscopic probe continues to hinder reliable identification of single atoms using HRTEM. As we shall see later in Section 3.3.1, this concern may have played an unappreciated role in the discovery of transmission electron microscopy based single-atom manipulation.

Before discussing atom manipulation, we need to consider another prominent family of atomic-resolution techniques, namely scanning probe microscopies. Although the challenges in identifying individual atoms are even greater in this case, until recently these were the only tool at our disposal to controllably manipulate them.

### 2.3 Scanning probe microscopies

Invented by Binnig and Röhrer in 1981, initially the probe in scanning tunneling microscopy (STM) was a sharp metallic tip, rastering over an atomically clean metallic surface while recording the tunneling current.^78^ Although STM gives excellent resolution due to the exponential dependence of the current on the tip-surface distance, this has no direct chemical sensitivity. Instead, the signal is proportional to the local density of electronic states (LDOS), which only with the aid of first-principles simulations can be interpreted as arising from specific atomic structures.

Alternatively, one can measure the force between an atomically sharp tip and the surface. This has the advantages of not requiring both (or either) to be metallic and enabling a plethora of interactions including frictional and magnetic forces to be used to form images, giving access to a much greater range of samples and surface properties. Dubbed atomic force microscopy (AFM), this technique was introduced by Binnig in 1986,^79^ although initially in ambient liquids so that atomic resolution was convincingly achieved by Giessibl only in 1996.^80^ An important further breakthrough was the discovery of molecular tip terminations, most prominent a vertically absorbed CO molecule, which greatly increased resolution and allowed even the distribution of electron density in the bonds of molecules to be resolved.^81^

Since the atom at the tip apex interacts with surface atoms altering its vibration frequency, different chemical elements can in some cases be distinguished. The elemental composition can potentially be recovered either *via* inelastic electron tunneling analysis of vibrational spectra^82^ or *via* careful comparison to first principles simulations of the LDOS, typically density functional theory (DFT).^83,84^ Identifying substitutional dopants is further possible in atomically thin materials, where the latter method has been used to study lattice heteroatoms in graphene^85^ and single-walled carbon nanotubes,^86^ although interpretation of the contrast can be challenging.^87^

## 3 Manipulating single atoms

Despite lacking chemical sensitivity, scanning probe microscopies, most prominently STM^88^ but also AFM,^89^ have been extremely capable tools for single-atom manipulation—and until recently, the only techniques capable of that feat. When an atomically clean surface can be prepared in an ultra-high vacuum and carefully controlled amounts of a known element are introduced, lack of direct chemical sensitivity is not a major issue. However, despite their many stunning successes, techniques based on scanning tips are obviously limited to surface atoms. Further, due to the limited interaction strength between the tip apex and the surface atoms, such manipulation almost invariably can only affect relatively weakly bound surface adatoms or vacancies, typically requiring the samples to be kept at cryogenic temperatures.

### 3.1 Pioneering scanning probe work and its limitations

The first successful atom manipulation was achieved by Eigler in 1990,^90^ using liquid helium (4 K) cryogenic STM to position xenon atoms on a single-crystal nickel surface with atomic precision. This was followed by iconic experiments in confining surface states by rings of atoms into so-called quantum corrals by Crommie in 1993.^91^ More recently, manipulation has been achieved at liquid nitrogen temperatures (77 K) by manipulating vacancies in surface monolayers. Combined with the automated operation, this has enabled feats such as the creation of a kilobyte atomic memory by Kalff in 2016,^92^ and significant recent effort has been directed at creating customized quantum states by atom manipulation of surfaces.^93^

Following the crucial development of non-contact imaging methods, AFM achieved atom manipulation only later,^94^ but is for specific materials able to operate even at room temperature. The required precise balance between a sufficiently small diffusion barrier to allow manipulation by a physical probe tip and a sufficiently high binding energy to avoid desorption was first achieved for molecules absorbed on metal surfaces,^95^ then for specific semiconductors,^94^ and finally also for insulating surfaces.^96^ More recently, simultaneous detection of forces and tunneling currents has blurred the boundaries between AFM and STM, yielding even richer information on the sample.^97^ Nonetheless, direct elemental identification remains arduous.

Manipulation with STM or AFM is strictly limited to surfaces since a tip must be brought into their immediate proximity, though embedded single-atom devices have been fabricated *via* hydrogen depassivation lithography by precisely placing dopant atoms on semiconductor surfaces followed by further semiconductor layer growth.^98^ While the room-temperature capabilities of AFM need to be acknowledged,^89^ the vast majority of published research uses STM and continues to require cryogenic temperatures, and thus any manipulated structures are ephemeral and only survive for as long as they are kept cool.

For these reasons, the discovery of scanning transmission electron microscopy-based manipulation techniques that operate at room temperature with strongly bound lattice impurities has opened exciting new possibilities, but also brought new tradeoffs and challenges.

### 3.2 New manipulation tool: focused electron irradiation

The focused electron beam of a modern STEM is an alternative atomically sharp probe, which can penetrate into the bulk of materials. However, before atom manipulation became possible, several technological preconditions had to be fulfilled. First, it is absolutely vital that the electron beam can be directed to an atomically small spot so that a desired atomic site can be selectively irradiated. Aberration correction of the probe-forming lenses was thus required, and it was also necessary to reach Å-sized probes even at sub-80 keV primary beam energies where undesirable knock-on damage can be suppressed. While current instruments are sufficient for this task, further reduction of the probe tails and an increase of the beam current would be highly beneficial.

Although somewhat less critical, the stability of the microscope sample stage, the reliability of the scan coils, and the suppression of unwanted chemical etching by a reduced residual vacuum pressure are all factors that can make the difference on whether manipulation is possible merely in theory, or actually feasible in practice. Arguably only the Nion microscopes fulfill all of these conditions fully, though certainly, these are engineering challenges that can be tackled by other manufacturers as well. In terms of suitable samples, several preconditions have to be fulfilled: atomically clean surfaces, sufficient stability under electron irradiation, the availability of impurities that can be easily identified, and crucially, suitable dynamics under electron irradiation.

Although single-atom dynamics had been observed earlier,^99^ I will focus on the historical development of STEM atom manipulation, which starts with silicon substitutions in graphene.

### 3.3 Silicon substitutions in graphene

Microscopists working with graphene inevitably have to grapple with surface contamination: any amount will ruin imaging of the one-atom-thick material, let alone more complicated experiments. Synthesis residues appear as thicker, possibly metal-containing contamination layers, whereas even brief ambient exposure can be sufficient to deposit a thin hydrocarbon layer. Accordingly, many kinds of cleaning strategies have been explored and employed.^100^ In addition to hydrocarbons, silicon is a ubiquitous contaminant in essentially all graphene samples, although the use of non-Si-containing glasswares for sample preparation does appear to reduce it.^101^ As another group IV element, Si has a high affinity to bond with C, and even substitute into the graphene lattice—indeed, such incidental impurities were the target of all early studies.^61,67,102^

Si either substitutes a single C atom in a three-coordinated configuration or instead binds with four neighbors replacing two C atoms. The former buckles out of the plane due to the larger covalent radius of Si compared to C, as originally elucidated *via* single-atom EELS interpreted in light of first-principles simulations^52^ and subsequently confirmed by a few-tilt tomographic reconstruction^53^ (Fig. 1). Si from the contamination can also be trapped in vacancies purposefully created using higher-energy electron irradiation,^103^ and more recently, vacancy-mediated substitution with Ar plasma has allowed us to fabricate better-controlled samples with a higher concentration of Si.^104^

However, the dynamics of the three-coordinated Si site under electron irradiation are the reason why it has played such an important role in the electron-beam manipulation research. With the benefit of hindsights, there were several studies that may constitute early hints of these dynamics. Indeed, I believe that before our foundational work in 2014,^67^ the beam-induced movement of Si impurities was observed several times since as early as 2008, although in each case with variously mistaken elemental identification.

#### 3.3.1 Early hints of single-atom dynamics

In a landmark study, in 2008, Meyer published the first atomically resolved images of graphene and its defects.^108^ This was soon followed by the apparent imaging of the dynamics of light atoms and molecules on its surface using phase-contrast HRTEM.^109^ However, considering the time required to capture such images (summed from up to 100 consecutive frames!), it seems obvious that the proposed atomic configurations—a carbon adatom in a bridge configuration and an oxygen adatom bound at a top site—can hardly be responsible for the observed contrast.

Even leaving aside the effect of the electron beam, which at 100 keV can easily transfer several eV of lateral kinetic energy to any light adatom,^110^ their sub-eV migration barriers are easy to overcome thermally at room temperature^111,112^ and adatoms cannot in fact be observed in atomically resolved images. Indeed, extreme care with irradiation dose needs to be taken even to capture the spectroscopic signatures of oxygen in graphene oxide, at vastly lower areal doses.^113^ It seems instead likely that what the authors observed were substitutional lattice impurities.

In a similar vein, in 2010, Erni used HRTEM to identify point contrast at graphene lattice positions as adsorbed O, N, or C-containing molecules.^105^ However, it seems equally implausible that multiple consecutive frames and focal series with electron doses of 10^6^ e^−^ nm^−2^ at 80 keV could be captured of beam-sensitive admolecules (H is particularly easily sputtered^114^), especially since isolated admolecules also exhibit sub-eV thermal diffusion barriers.^115^ No spectroscopic evidence was available in either of these studies.

**Fig. 2:**
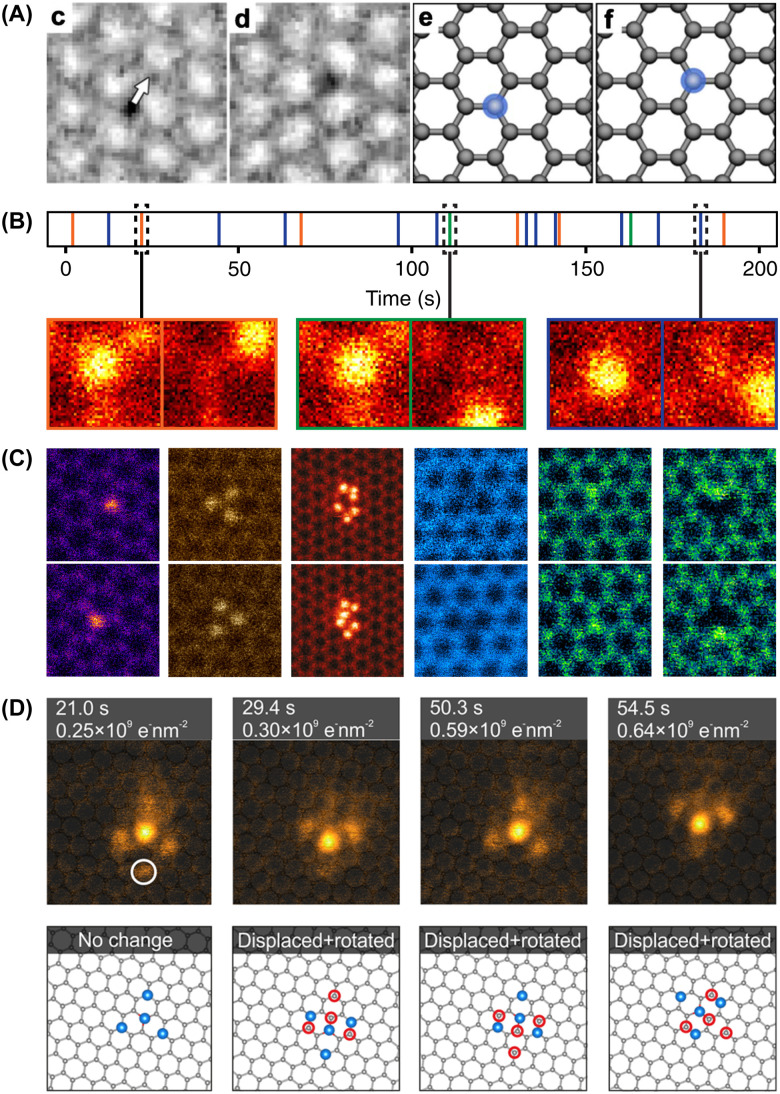
Dynamics of single atoms observed with transmission electron microscopy. (A) Two consecutive HRTEM phase-contrast images of what was identified as an admolecule binding over a carbon atom in graphene, moving from one lattice site to the next under 80 keV electron irradiation. Adapted with permission from ref. 105. Copyright (2010) by the American Physical Society. (B) Time series of 19 consecutive jumps of an EELS-verified Si impurity in graphene recorded with STEM at 60 keV. Adapted with permission from ref. 67 (CC-BY 3.0). (C) Atom-conserving beam-induced dynamics of various impurity atoms in graphene (top and bottom show consecutive ADF frames), from left to right: substitutional Si, Si trimer in a hexavacancy, Si_6_ cluster in a pore, substitutional B, substitutional N, pyridinic N in a single vacancy. Reproduced with permission from ref. 106 (CC-BY 3.0). (D) A four-atom In cluster bound to a three-coordinated Si substitution in graphene undergoing rotation and translation under 60 keV electron irradiation. Reproduced with permission from ref. 107 (CC-BY 4.0).

I suspect the latter authors may actually have been the first to detect the motion of Si impurities within the lattice under electron irradiation:^105^ “Although the positions of defects 1 and 2 remain unchanged, the position of defect 3 changes: it moves from one carbon atom to a neighboring carbon position.” They took this as evidence against lattice substitutions:^105^ “Since a correlated diffusion-based exchange of a substitutional atom with a graphene carbon atom is unlikely to occur at room temperature without having a vacancy involved, we conclude that the defects […] are due to atoms attached to the graphene lattice” (see Fig. 2B). In fact, we now know that precisely this happens to electron-irradiated Si impurities in graphene.

**Fig. 3:**
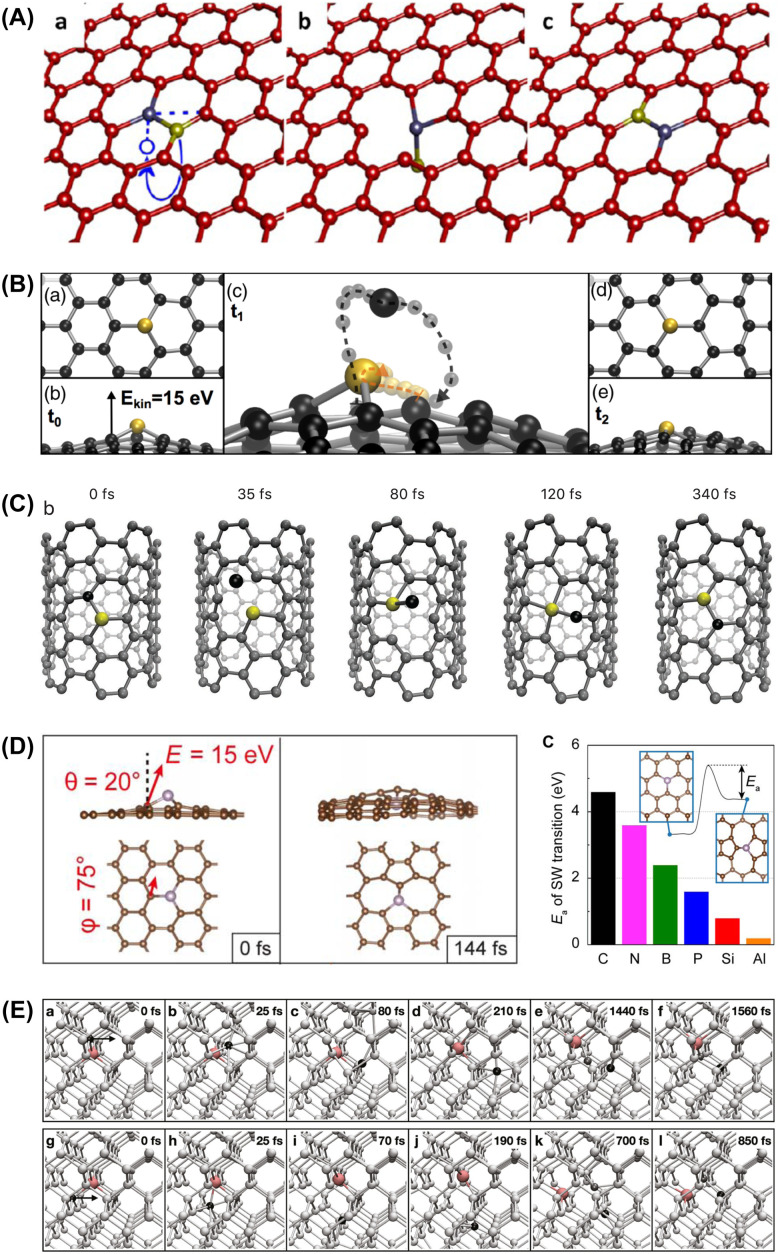
Mechanisms of non-damaging dynamics of single impurity atoms under electron irradiation simulated with density functional theory molecular dynamics. (A) The exchanging of places between an Fe substitution in graphene (gray blue) and one of the three C neighbors (yellow highlight) being transiently ejected due to an out-of-plane kinetic energy transfer. Reproduced with permission from ref. 65. (B) Direct exchange (bond inversion) of a three-coordinated Si impurity with one of its three C neighbors due to the out-of-plane dynamics following a 15 eV kinetic energy transfer. Simulation time *t*_1_ is 70 fs and *t*_2_ is 140 fs. Reproduced with permission from ref. 67 (CC-BY 3.0). (C) Direct exchange of a three-coordinated Si impurity within the lattice of a narrow armchair single-walled carbon nanotube. Reproduced with permission from ref. 116 (CC-BY 4.0). (D) Kinetic energy transfer at a 20° angle with respect to the surface normal leading to the bond rotation at a P substitution in graphene, and the corresponding simulated activation energies for different impurity elements. Adapted with permission from ref. 71. (E) Indirect exchange of a Bi dopant (pink) in crystalline silicon (pale grey) induced by a kinetic energy transfer of 14.5 eV in the^110^ lattice direction (black arrows) on either of the two Si neighbors (top and bottom rows, black highlight). Reproduced with permission from ref. 117 (CC-BY 4.0).

Such an exchange was proposed in 2012 by Robertson, who presented HRTEM images of what they thought to be three- and four-coordinated iron substitutions in graphene samples with FeCl_3_ residues.^65^ Indirect large-area EELS showed the presence of Si and C in addition to Fe in the sample, and supplementary ADF-STEM and EELS data confirmed the presence of Fe impurities at pore edges. However, despite comparisons to image simulations—which I would argue were overly idealized in terms of coherence and noise—it is not clear whether the substitutional impurities in the lattice only imaged by HRTEM were in fact Fe, as no ADF-STEM or spectra on them was presented. Notably, in other studies with ADF images and spectra of confirmed Fe single atoms, these are found as four-coordinated substitutions in divacancies.^57,60,69^ Nonetheless, the dynamical mechanism they presented for Fe is strikingly similar to the mechanism for Si manipulation that we uncovered (see Fig. 3A and B).

In all of these cases, ADF-STEM would have been sufficient to conclusively identify the impurities, although single-atom EELS should be considered the ideal standard in terms of evidence.

#### 3.3.2 Beam-induced dynamics and their control

Thus, although the dynamics of Si impurities in graphene were arguably observed already in 2010, if not earlier, and a qualitative description of such dynamics was proposed for Fe in 2013, I was not aware of these studies when we published our work on Si in 2014 (submitted on 3 March 2014, released on arXiv on 16 July 2014, and published on 11 September 2014).^67^ In our study, we provided single-atom EELS data to conclusively identify the impurity atoms as Si and a quantitative model of the direct exchange mechanism for their movement: a 60-keV electron can give one of the three C neighbors of Si a “kick” *via* elastic backscattering, causing it to almost but not quite eject from the lattice. During its out-of-plane dynamics, Si relaxes into the lattice position vacated by the ejecting C, which then gets recaptured into the lattice but on the opposite side of its original position. Effectively, Si has moved by one lattice site, with no atoms lost from the structure—contrary to what Erni and colleagues had expected.^105^

Most importantly, we appear to have been the first to explicitly draw the conclusion that Si can be manipulated with atomic precision using the electron beam: by purposefully focusing the irradiation onto one selected C neighbor of Si, the direction of its movement can be controlled. This key finding was highlighted in the September 2014 issue of *Physics Today*.^118^ However, experimental proof of manipulation was not actually reported until more than two years later.

#### 3.3.3 Manipulation of silicon impurities

**Fig. 4:**
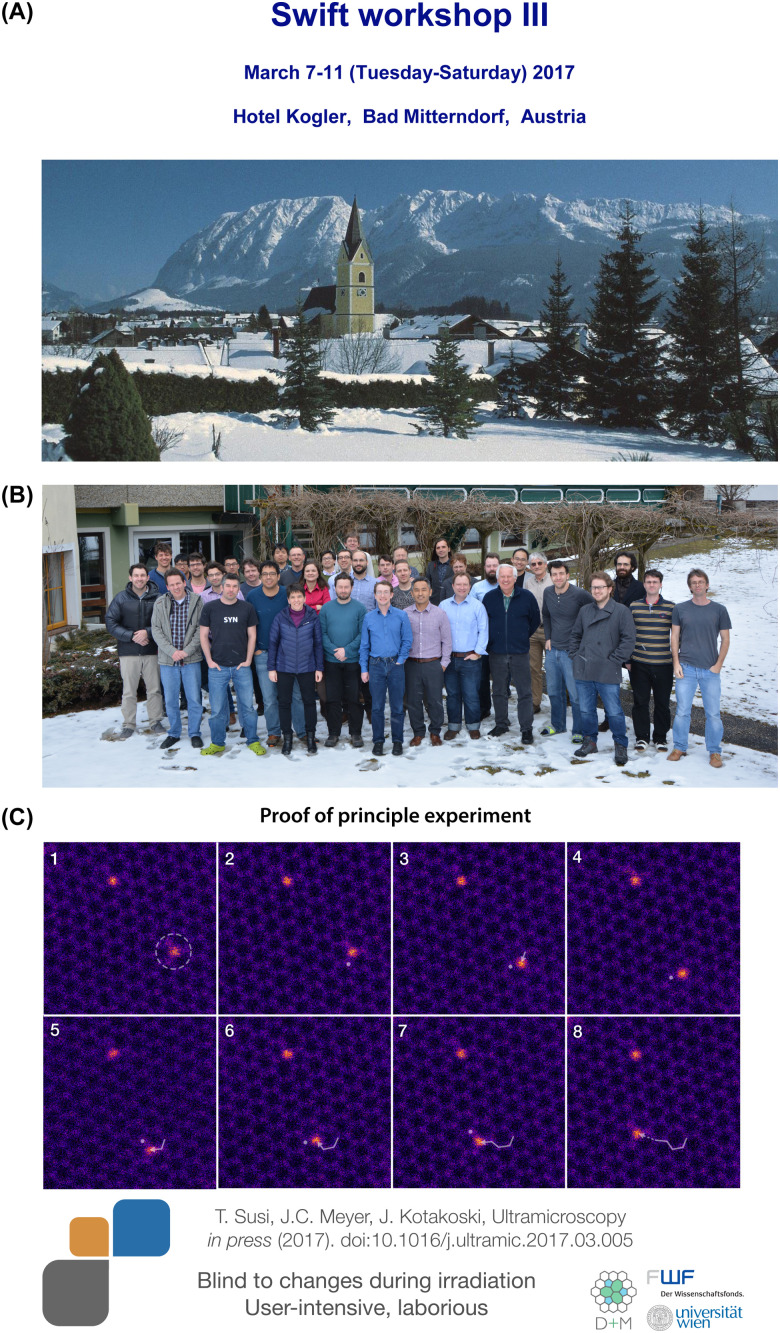
(A) Cover of the third Nion Swift workshop (courtesy of Nion Co). (B) Group photo of the participants (courtesy of A. Kogler^120^). (C) Slide of the first controlled electron-beam manipulation of a Si impurity in graphene the author presented at the meeting.

**Fig. 5:**
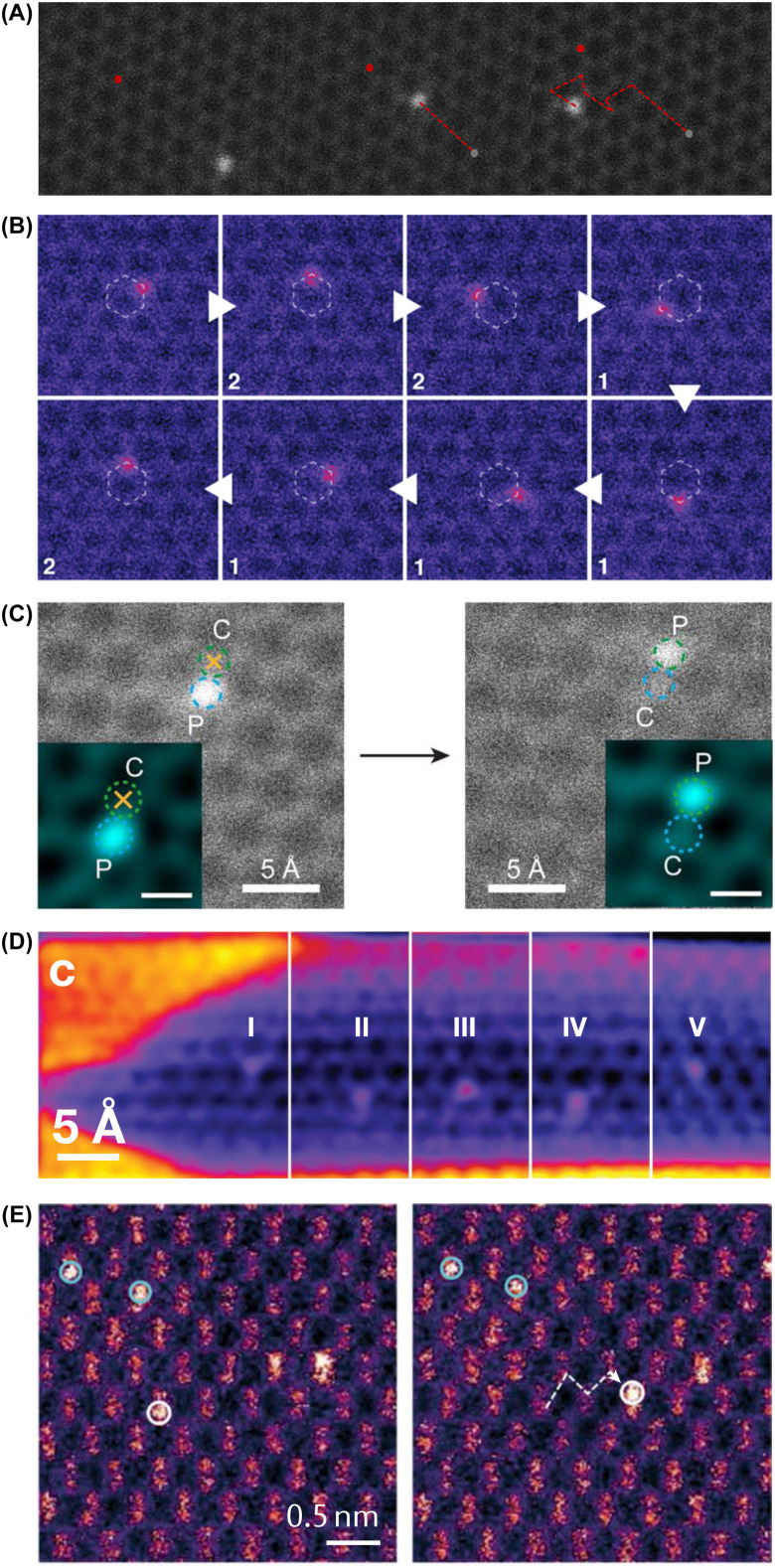
Manipulation of impurity atoms (ADF-STEM images). (A) First independent replication of Si manipulation in graphene at 60 keV. Adapted with permission from ref. 103, with the permission of AIP Publishing. (B) High control over the movement of Si around a graphene hexagon at 60 keV. Overlaid are the number of 10 second spot irradiations of the C sites where Si was moved. Reproduced with permission from ref. 121 (CC-BY 4.0). (C) Direct exchange of a P impurity in graphene induced by 60 keV spot irradiation on the C site marked by the yellow cross. The insets show filtered images. Reproduced with permission from ref. 71 (CC-BY 4.0). (D) Selected frames from an ADF image sequence showing the manipulation of Si along the axis of a single-walled carbon nanotube at 60 keV. Reproduced with permission from ref. 116 (CC-BY 4.0). (E) Manipulation of a Bi dopant (white circle) within a crystalline silicon lattice using rapid 200 keV spot irradiations along the desired path (white dashed line). Reproduced with permission from ref. 137. Copyright 2018 American Chemical Society.

After successfully applying for funding to pursue this research (the proposal was published in 2015)^119^ and delays due to instrument upgrades, we conducted the first manipulation experiments using a Nion UltraSTEM100 in Vienna over the summer of 2016. The results were submitted to Ultramicroscopy on September 30, 2016, and after a revision on February 24, 2017, published on March 2, 2017. I presented them at the Nion Swift workshop in Austria on March 7–11, 2017 (Fig. 4), explaining how the manipulation is done in practice by parking the electron beam at a chosen C neighbor between acquiring image frames. The meeting was attended by colleagues from the USA and was soon followed by the first independent replication by Dyck (submitted on August 1, 2017 and published on September 14, 2017).^103^ Notably, they used a small subscan window on a selected C neighbor (Fig. 5A), which arguably resulted in poorer control, but likely is more amenable for active drift compensation.

We reported significantly improved control in June 2018, including lowering the beam energy to 55 keV and implementing real-time feedback from the ADF signal.^121^ However, despite the obvious interest and theoretical predictions,^122^ multi-atom structures remain a challenge to this day. Incidental Si structures in graphene pores have been reported,^123–125^ including 2D silicon-carbide,^126^ as well as partially controlled dynamics of imperfect multi-Si configurations^127^ (the tendency for Si to cluster was noted based on simulations in our original publication^67^). A major practical challenge for scaling up the method is an unknown chemical process, whereby Si and other atom impurities get replaced by C during observation or manipulation,^71,73,116^ presumably due to the thermal diffusion of C adatoms.^112^

As Si was the first impurity to be successfully manipulated, it is by far the most studied element. Thanks to their similarity to graphene, in 2019, we were also able to demonstrate manipulation of Si in single-walled carbon nanotubes,^116^ and more recently found that in graphene, they can act as atomically precise anchors for few-atom In clusters while retaining the possibility of movement through the lattice^107^ (Fig. 2D). However, other impurities that do not share the same valence as C are of great potential interest. Accordingly, increasing efforts have been directed at fabricating suitable samples, studying the dynamics of the dopants, and in limited cases, even demonstrating that manipulation is possible. There is also interest in extending the technique to other materials, although these present unique challenges. Table 2 lists all of the published examples of manipulation in graphene as well as other materials, which we will turn to discuss next.

**Table 2:** Electron-beam dynamics of individual impurity atoms and dopants, and their atomically precise manipulation in graphene and other materials. Results include observation of dynamics (D), theoretical description of the mechanism (T), and manipulation (M)

Material	Element	Results	Note	Ref.
Graphene	Fe	D, T	Actually Si?	65 and 128
	Si	D, T	M proposed	67
	Si	M	M demonstrated	134
	Si	D, M		103, 121 and 127
	N, B	D		106
	N	D, T		135
	P	D, T, M		71
	Al	D, T		40
SWCNT	Si	T, M	Near axis	116
MoS_2_	Re	D, T, M	Damaging	131
silicon	Bi	D, M	M in 3D	136 and 137
	Bi, Sb	T, M	Novel mechanism	117

### 3.4 Other dopants in graphene

Besides silicon impurities, only phosphorus dopants have been experimentally manipulated, as we reported in 2019 together with Su.^71^ However, the competing process of replacement by C was an even more serious problem here, and only two controlled jumps could be shown. Modeling of the manipulation mechanism showed that the energy window where the dopant can be moved but a C neighbor is not knocked out is rather narrow for P. On the other hand, Al substitution was predicted to be highly amenable for manipulation,^71^ which was partially confirmed by Zagler in 2022, who observed their non-destructive dynamics.^40^ However, purposeful manipulation of Al has not yet been shown.

There also seems to be a limit on how heavy elements can still be manipulated with the direct exchange mechanism: the impurity has to move into the transient vacancy created by the C atom displaced by the electron impact before it lands back into the lattice. This is not the case for germanium, which first-principles modeling revealed does not move sufficiently swiftly,^73^ explaining why they could not be manipulated. The same issue very likely applies to other heavier elements such as platinum^56,74^ and gold,^76^ which have not been observed to move under irradiation. Further, the impurity ideally needs to be stable in a buckled three-coordinated configuration, which is not typically the case for many metals.^75^ However, simulations have shown the possibility to manipulate transition metals including Fe,^128^ which could be very interesting for tunable local magnetic properties.^57,72^

At the lighter end, we have shown that both nitrogen and boron can move within the lattice without damage.^106^ However, the required doses are much higher than for Si at the same electron energy, and the theoretical mechanism is not yet clear. Since these dopants do not buckle out of the plane, the same direct exchange mechanism *via* an impact on a carbon neighbor does not result in it switching places with the dopant. Potentially, the mechanism here is instead *via* two bond rotations in quick succession^71^ (Fig. 3D). Purposeful manipulation has likewise not yet been demonstrated, and experiments on B and N are more challenging due to the relative lack of samples and the low contrast of these elements with respect to carbon with ADF imaging.

### 3.5 Dopants in other 2D materials

Graphene is not ideal for many applications due to its metallic nature, and thus, the possibility to manipulate dopants in other 2D materials is a question of obvious interest. However, this metallicity is also what gives graphene its extremely high electron irradiation tolerance at primary beam energies below 80 keV.^129^ Since electron-beam manipulation uses the energetic electron beam to both image and manipulate, it is crucial that damage or other unwanted changes in the structure can be eliminated or at least mitigated. Unfortunately, the most common alternative 2D materials, such as the semiconducting MoS_2_ or the insulating hBN, appear to damage *via* a combination of inelastic excitations and knock-on damage.^130^ Thus, there is no “safe” beam energy that can be selected that precludes damage to the host material, making such experiments daunting.

Accordingly, there are, to the best of my knowledge, still no published examples of atomically precise manipulation in other 2D materials than graphene. The closest to this comes to the work by Yang in 2019,^131^ who observed the vacancy-mediated movement of Re impurities through the MoS_2_ lattice. The authors could demonstrate some control over the direction of their movement, although only at the cost of creating vacancies along the path. Conceivably, metallic 2D transition metal dichalcogenides,^132^ such as TaS_2_, NbSe2, or MoTe_2_, or other metallic 2D materials,^133^ could prove suitable for manipulation, but no simulations of atomistic mechanisms or experimental studies have yet been reported. However, another widely studied bulk system has proven surprisingly amenable for electron-beam atom manipulation, with potentially exciting applications that can be envisioned, namely crystalline silicon.

### 3.6 Dopants in crystalline silicon

A significant limitation of electron-beam manipulation is that there is a competing mechanism to the controlled movement of the atoms through the lattice: irradiation-induced sputtering. This leads to the removal of either one of the lattice atoms or the impurity atom after a stochastically determined number of steps that depends on the electron energy.^121^ However, a similar manipulation process has also been demonstrated for Bi^137^ and Sb^117^ dopants in bulk silicon, which may prove more robust due to the 3D lattice. The contrast of lighter dopants would present a major challenge, but it also appears that the same mechanism does not work for P and As^117^—highlighting the value of simulations in guiding experiments.

### 3.7 Manipulation mechanisms

As already hinted at above, manipulation mechanisms can be divided into two categories: non-damaging, where the number of atoms is conserved and which has been the main focus of my work, and damaging, which involves the loss of atoms from the material. Purposefully creating vacancies with the electron beam to fill them with impurities straddles the divide between the two categories,^103^ and several reviews on sculpting and manipulating structures with electron beams are available for the reader interested in the latter.^134,138–140^

In terms of non-damaging mechanisms, essentially two kinds have been identified (Fig. 3). The first we originally dubbed “bond inversion”^67^ but more recently call “direct exchange”.^71^ It involves kinetic energy transfer to an atom neighboring an impurity, which then in a dynamical trajectory directly exchanges places with it. A two-step variant can in graphene occur *via* an intermittent Stone-Wales bond rotation.^71,141^ It is also possible that some observed dynamics include a transient adatom, as we recently simulated for the pyridinic N defect in graphene.^135^

The second type of process was identified for the manipulation of substitutional dopants in crystalline silicon,^137^ which involves a more complex coordinated “dance” of the atoms around the impurity. Here, the host lattice atom originally neighboring the impurity ends up as its second-nearest neighbor, with another lattice atom taking over the position originally occupied by the impurity atom. We therefore named this process “indirect exchange”, where, crucially, vacancies do not need to be created.^117^

However, it should be mentioned that these studies have only considered kinetic energy transfers due to elastic scattering, which is all that it has been possible to accurately model. Not only is it clear that this is not sufficient to describe electron irradiation effects in materials that are not metals (including hBN and MoS_2_, as discussed in our recent review^130^), we have recently shown that not even the beam-induced dynamics of impurity sites in graphene can be correctly quantified by purely elastic models.^106,135^ While very recent theoretical advances have started to include inelastic excitations,^142–144^ significant further work is needed before we can reliably understand the role they might play in atom manipulation.

To expand the possibilities of electron-beam manipulation to new impurities and new materials, novel mechanisms may need to be discovered and exploited. However, it seems safe to predict that nature has more surprises in store for us yet.

## 4 Conclusion and outlook

The use of focused electron irradiation to manipulate single atoms has developed from a theoretical possibility to a viable technique in several materials over the past eight years. While much progress has been made, the challenges that remain will ultimately determine how far we can expect to advance. The most immediate hurdle is the availability of clean samples with a high concentration of substitutional impurities with the right kind of bonding. Despite clever sample preparation and cleaning techniques, the effort required as well as the limited reproducibility and availability of samples do hinder progress.

From a personal point of view, and with the privilege of working in a laboratory that houses a unique interconnected ultra-high vacuum sample preparation and characterization apparatus,^145^ our own work has been most severely affected by unwanted interactions. In most cases, knock-on damage competes with the desired non-damaging dynamics, but even more severely, the replacement of impurities in graphene with carbon has prevented us from creating larger patterns. The mechanism for this remains unknown, though diffusing adatoms are certainly involved and heating to reasonable temperatures does not help;^112^ however, stable cryogenic stages could be a crucial innovation. Should these challenges be overcome, the increasing trend towards data-driven and automated transmission electron microscopy appears poised to enable rapid progress.^146^ Automated atom detection using neural networks^147^ running in real-time on the microscope^148^ and combined with real-time feedback of the scattering signal^121,136^ are enabling more than one group to build fully automated manipulation software.^149,150^ A self-driving electron microscope^151^ appears to be finally within reach.

Using focused electron irradiation in a scanning transmission electron microscope instead of a physical tip in a scanning probe microscope can confer several benefits. These include the thermal stability of the manipulated structures due to the higher interaction energy available to alter strong covalent bonds, the ability to reach into bulk crystals due to the transmission of the electrons through the specimen, and the unambiguous chemical identification of single atoms. Scanning electron beams can also be controlled at a higher throughput than physically scanning tips, though parallelization of manipulations will require further breakthroughs in aberration corrector miniaturization and instrument design.

I am excited to further contribute to these developments and see how far we will be able to push the limits of physics and technology—in twenty years, we may look back on this era as a historical curiosity, or as the start of something greater.

## Conflicts of interest

Although no financial incentives are involved, the author has an interest in establishing scientific priority for his contributions. To the best of his ability, any statements made in the text are based on documented and verifiable facts and dates.

## Supplementary Material

## References

[cit1] Joy L. S. , Gassendi the Atomist: Advocate of History in an Age of Science, Cambridge University Press, Cambridge, 1988

[cit2] Dalton J. , A New System of Chemical Philosophy, ed. S. Russell, 1808

[cit3] Thackray A. W. (1966). Isis.

[cit4] Cameron F. K. (1900). Science.

[cit5] Tang K.-T., Toennies J. P. (2010). Angew. Chem., Int. Ed..

[cit6] Toulmin S. , Physical reality; philosophical essays on twentieth-century physics, Harper & Row, New York, 1970

[cit7] Planck M. (1896). Ann. Phys..

[cit8] Einstein A. (1905). Ann. Phys..

[cit9] Perrin J. (1909). Ann. Chim. Phys..

[cit10] Thomson J. J. (1897). London, Edinburgh Dublin Philos. Mag. J. Sci..

[cit11] Rutherford E. (1911). London, Edinburgh Dublin Philos. Mag. J. Sci..

[cit12] Soddy F. (1913). Nature.

[cit13] Müller E. W., Bahadur K. (1956). Phys. Rev..

[cit14] Tsong T. T. (2001). Prog. Surf. Sci..

[cit15] Katnagallu S., Stephenson L. T., Mouton I., Freysoldt C., Subramanyam A. P. A., Jenke J., Ladines A. N., Neumeier S., Hammerschmidt T., Drautz R., Neugebauer J., Vurpillot F., Raabe D., Gault B. (2019). New J. Phys..

[cit16] Müller E. W., Panitz J. A., McLane S. B. (1968). Rev. Sci. Instrum..

[cit17] Gault B., Chiaramonti A., Cojocaru-Mirédin O., Stender P., Dubosq R., Freysoldt C., Makineni S. K., Li T., Moody M., Cairney J. M. (2021). Nat. Rev. Methods Primers.

[cit18] Thomson G. P., Reid A. (1927). Nature.

[cit19] Davisson C. J., Germer L. H. (1928). Proc. Natl. Acad. Sci. U. S. A..

[cit20] Ruska E. , The Early Development of Electron Lenses and Electron Microscopy, Hirzel, 1980 6997691

[cit21] Crewe A. V., Wall J., Langmore J. (1970). Science.

[cit22] Treacy M. M. J., Rice S. B. (1989). J. Microsc..

[cit23] Nellist P. D., Pennycook S. J. (1996). Science.

[cit24] Voyles P. M., Muller D. A., Grazul J. L., Citrin P. H., Gossmann H.-J. L. (2002). Nature.

[cit25] Wang S., Borisevich A. Y., Rashkeev S. N., Glazoff M. V., Sohlberg K., Pennycook S. J., Pantelides S. T. (2004). Nat. Mater..

[cit26] van Benthem K., Lupini A. R., Kim M., Baik H. S., Doh S., Lee J.-H., Oxley M. P., Findlay S. D., Allen L. J., Luck J. T., Pennycook S. J. (2005). Appl. Phys. Lett..

[cit27] Iijima S. (1971). J. Appl. Phys..

[cit28] Gan Y., Sun L., Banhart F. (2008). Small.

[cit29] Meyer J. C., Kurasch S., Park H. J., Skakalova V., Künzel D., GroÃŸ A., Chuvilin A., Algara-Siller G., Roth S., Iwasaki T., Starke U., Smet J. H., Kaiser U. (2011). Nat. Mater..

[cit30] Urban K. W., Mayer J., Jinschek J. R., Neish M. J., Lugg N. R., Allen L. J. (2013). Phys. Rev. Lett..

[cit31] Ishikawa R., Okunishi E., Sawada H., Kondo Y., Hosokawa F., Abe E. (2011). Nat. Mater..

[cit32] de Graaf S., Momand J., Mitterbauer C., Lazar S., Kooi B. J. (2020). Sci. Adv..

[cit33] Lin B., Wu X., Xie L., Kang Y., Du H., Kang F., Li J., Gan L. (2020). Angew. Chem., Int. Ed..

[cit34] Zhu Y., Inada H., Nakamura K., Wall J. (2009). Nat. Mater..

[cit35] Inada H., Su D., Egerton R., Konno M., Wu L., Ciston J., Wall J., Zhu Y. (2011). Special Issue, J. Spence's 65th birthday.

[cit36] Hawkes P. W. (2009). Philos. Trans. R. Soc. London, Ser. A.

[cit37] Krivanek O. , Dellby N., Spence A., Camps R. and Brown L., Inst. Phys. Conf. Ser. 153 (Proceedings 1997 EMAG meeting), 1997, pp. 35–40

[cit38] Haider M., Uhlemann S., Schwan E., Rose H., Kabius B., Urban K. (1998). Nature.

[cit39] Krivanek O. L., Chisholm M. F., Nicolosi V., Pennycook T. J., Corbin G. J., Dellby N., Murfitt M. F., Own C. S., Szilagyi Z. S., Oxley M. P., Pantelides S. T., Pennycook S. J. (2010). Nature.

[cit40] Zagler G., Stecher M., Trentino A., Kraft F., Su C., Postl A., Längle M., Pesenhofer C., Mangler C., Harriet Åhlgren E., Markevich A., Zettl A., Kotakoski J., Susi T., Mustonen K. (2022). 2D Mater..

[cit41] Colliex C., Gloter A., March K., Mory C., Stéphan O., Suenaga K., Tencé M. (2012). Ultramicroscopy.

[cit42] Batson P. E. (1993). Nature.

[cit43] Browning N. D., Chisholm M. F., Pennycook S. J. (1993). Nature.

[cit44] Egerton R. F. (2009). Rep. Prog. Phys..

[cit45] Kimoto K., Asaka T., Nagai T., Saito M., Matsui Y., Ishizuka K. (2007). Nature.

[cit46] Bosman M., Keast V. J., García-Muñoz J. L., DAlfonso A. J., Findlay S. D., Allen L. J. (2007). Phys. Rev. Lett..

[cit47] Gunawan A. A., Mkhoyan K. A., Wills A. W., Thomas M. G., Norris D. J. (2011). Nano Lett..

[cit48] Rossell M. D., Ramasse Q. M., Findlay S. D., Rechberger F., Erni R., Niederberger M. (2012). ACS Nano.

[cit49] DAlfonso A. J., Freitag B., Klenov D., Allen L. J. (2010). Phys. Rev. B: Condens. Matter Mater. Phys..

[cit50] Suenaga K., Tencé M., Mory C., Colliex C., Kato H., Okazaki T., Shinohara H., Hirahara K., Bandow S., Iijima S. (2000). Science.

[cit51] Varela M., Findlay S. D., Lupini A. R., Christen H. M., Borisevich A. Y., Dellby N., Krivanek O. L., Nellist P. D., Oxley M. P., Allen L. J., Pennycook S. J. (2004). Phys. Rev. Lett..

[cit52] Ramasse Q. M., Seabourne C. R., Kepaptsoglou D.-M., Zan R., Bangert U., Scott A. J. (2013). Nano Lett..

[cit53] Hofer C., Skakalova V., Monazam M. R. A., Mangler C., Kotakoski J., Susi T., Meyer J. C. (2019). Appl. Phys. Lett..

[cit54] Hage F. S., Radtke G., Kepaptsoglou D. M., Lazzeri M., Ramasse Q. M. (2020). Science.

[cit55] Suenaga K., Okazaki T., Okunishi E., Matsumura S. (2012). Nat. Photonics.

[cit56] Lovejoy T. C., Ramasse Q. M., Falke M., Kaeppel A., Terborg R., Zan R., Dellby N., Krivanek O. L. (2012). Appl. Phys. Lett..

[cit57] Lin Y.-C., Teng P.-Y., Chiu P.-W., Suenaga K. (2015). Phys. Rev. Lett..

[cit58] Langer R., Mustonen K., Markevich A., Otyepka M., Susi T., Błoński P. (2022). ACS Appl. Nano Mater..

[cit59] Senga R., Suenaga K. (2015). Nat. Commun..

[cit60] Chisholm M. F., Duscher G., Windl W. (2012). Nano Lett..

[cit61] Zhou W., Lee J., Nanda J., Pantelides S. T., Pennycook S. J., Idrobo J.-C. (2012). Nat. Nanotechnol..

[cit62] Bangert U., Pierce W., Kepaptsoglou D. M., Ramasse Q., Zan R., Gass M. H., Van den Berg J. A., Boothroyd C. B., Amani J., Hofsäss H. (2013). Nano Lett..

[cit63] Susi T., Hardcastle T. P., Hofsäss H., Mittelberger A., Pennycook T. J., Mangler C., Drummond-Brydson R., Scott A. J., Meyer J. C., Kotakoski J. (2017). 2D Mater..

[cit64] Wang H., Wang Q., Cheng Y., Li K., Yao Y., Zhang Q., Dong C., Wang P., Schwingenschlögl U., Yang W., Zhang X. X. (2012). Nano Lett..

[cit65] Robertson A. W., Montanari B., He K., Kim J., Allen C. S., Wu Y. A., Olivier J., Neethling J., Harrison N., Kirkland A. I., Warner J. H. (2013). Nano Lett..

[cit66] He Z., He K., Robertson A. W., Kirkland A. I., Kim D., Ihm J., Yoon E., Lee G.-D., Warner J. H. (2014). Nano Lett..

[cit67] Susi T., Kotakoski J., Kepaptsoglou D., Mangler C., Lovejoy T. C., Krivanek O. L., Zan R., Bangert U., Ayala P., Meyer J. C., Ramasse Q. (2014). Phys. Rev. Lett..

[cit68] Warner J. H., Lin Y.-C., He K., Koshino M., Suenaga K. (2014). ACS Nano.

[cit69] Deng D., Chen X., Yu L., Wu X., Liu Q., Liu Y., Yang H., Tian H., Hu Y., Du P., Si R., Wang J., Cui X., Li H., Xiao J., Xu T., Deng J., Yang F., Duchesne P. N., Zhang P., Zhou J., Sun L., Li J., Pan X., Bao X. (2015). Sci. Adv..

[cit70] Qiu H.-J., Ito Y., Cong W., Tan Y., Liu P., Hirata A., Fujita T., Tang Z., Chen M. (2015). Angew. Chem., Int. Ed..

[cit71] Su C., Tripathi M., Yan Q.-B., Wang Z., Zhang Z., Hofer C., Wang H., Basile L., Su G., Dong M., Meyer J. C., Kotakoski J., Kong J., Idrobo J.-C., Susi T., Li J. (2019). Sci. Adv..

[cit72] Langer R., Błoński P., Hofer C., Lazar P., Mustonen K., Meyer J. C., Susi T., Otyepka M. (2020). ACS Appl. Mater. Interfaces.

[cit73] Tripathi M., Markevich A., Böttger R., Facsko S., Besley E., Kotakoski J., Susi T. (2018). ACS Nano.

[cit74] Dyck O., Zhang C., Rack P. D., Fowlkes J. D., Sumpter B., Lupini A. R., Kalinin S. V., Jesse S. (2020). Carbon.

[cit75] Dyck O., Zhang L., Yoon M., Swett J. L., Hensley D., Zhang C., Rack P. D., Fowlkes J. D., Lupini A. R., Jesse S. (2021). Carbon.

[cit76] Trentino A., Mizohata K., Zagler G., Längle M., Mustonen K., Susi T., Kotakoski J., Åhlgren E. H. (2022). 2D Mater..

[cit77] Ullah S., Liu Y., Hasan M., Zeng W., Shi Q., Yang X., Fu L., Ta H. Q., Lian X., Sun J., Yang R., Liu L., Rümmeli M. H. (2022). Nano Res..

[cit78] Binnig G., Rohrer H., Gerber C., Weibel E. (1982). Phys. Rev. Lett..

[cit79] Binnig G., Quate C. F., Gerber C. (1986). Phys. Rev. Lett..

[cit80] Giessibl F. J. (1995). Science.

[cit81] Gross L., Mohn F., Moll N., Liljeroth P., Meyer G. (2009). Science.

[cit82] Stipe B. C., Rezaei M. A., Ho W. (1998). Science.

[cit83] Hofer W. A., Ritz G., Hebenstreit W., Schmid M., Varga P., Redinger J., Podloucky R. (1998). Surf. Sci..

[cit84] Sugimoto Y., Pou P., Abe M., Jelinek P., Pérez R., Morita S., Custance Ó. (2007). Nature.

[cit85] Zhao L., He R., Rim K. T., Schiros T., Kim K. S., Zhou H., Gutiérrez C., Chockalingam S. P., Arguello C. J., Pálová L., Nordlund D., Hybertsen M. S., Reichman D. R., Heinz T. F., Kim P., Pinczuk A., Flynn G. W., Pasupathy A. N. (2011). Science.

[cit86] Czerw R., Terrones M., Charlier J., Blase X., Foley B., Kamalakaran R., Grobert N., Terrones H., Tekleab D., Ajayan P., Blau W., Ruhle M., Carroll D. (2001). Nano Lett..

[cit87] Zheng B., Hermet P., Henrard L. (2010). ACS Nano.

[cit88] Stroscio J. A., Eigler D. M. (1991). Science.

[cit89] Custance Ó., Pérez R., Morita S. (2009). Nat. Nanotechnol..

[cit90] Eigler D. M., Schweizer E. K. (1990). Nature.

[cit91] Crommie M. F., Lutz C. P., Eigler D. M. (1993). Science.

[cit92] Kalff F. E., Rebergen M. P., Fahrenfort E., Girovsky J., Toskovic R., Lado J. L., Fernández-Rossier J., Otte A. F. (2016). Nat. Nanotechnol..

[cit93] Khajetoorians A. A., Wegner D., Otte A. F., Swart I. (2019). Nat. Rev. Phys..

[cit94] Sugimoto Y., Abe M., Hirayama S., Oyabu N., Custance Ó., Morita S. (2005). Nat. Mater..

[cit95] Jung T. A., Schlittler R. R., Gimzewski J. K., Tang H., Joachim C. (1996). Science.

[cit96] Kawai S., Foster A. S., Canova F. F., Onodera H., Kitamura S.-i, Meyer E. (2014). Nat. Commun..

[cit97] Sawada D., Sugimoto Y., Morita K.-i, Abe M., Morita S. (2009). Appl. Phys. Lett..

[cit98] Fuechsle M., Miwa J. A., Mahapatra S., Ryu H., Lee S., Warschkow O., Hollenberg L. C. L., Klimeck G., Simmons M. Y. (2012). Nat. Nanotechnol..

[cit99] Mishra R., Ishikawa R., Lupini A. R., Pennycook S. J. (2017). MRS Bull..

[cit100] Tripathi M., Mittelberger A., Mustonen K., Mangler C., Kotakoski J., Meyer J. C., Susi T. (2017). Phys. Status Solidi RRL.

[cit101] Butz B., Dolle C., Halbig C. E., Spiecker E., Eigler S. (2016). Angew. Chem., Int. Ed..

[cit102] Ramasse Q. M., Zan R., Bangert U., Boukhvalov D. W., Son Y.-W., Novoselov K. S. (2012). ACS Nano.

[cit103] Dyck O., Kim S., Kalinin S. V., Jesse S. (2017). Appl. Phys. Lett..

[cit104] Inani H., Mustonen K., Markevich A., Ding E.-X., Tripathi M., Hussain A., Mangler C., Kauppinen E. I., Susi T., Kotakoski J. (2019). J. Phys. Chem. C.

[cit105] Erni R., Rossell M. D., Nguyen M.-T., Blankenburg S., Passerone D., Hartel P., Alem N., Erickson K., Gannett W., Zettl A. (2010). Phys. Rev. B: Condens. Matter Mater. Phys..

[cit106] Susi T., Kepaptsoglou D., Lin Y.-C., Ramasse Q., Meyer J. C., Suenaga K., Kotakoski J. (2017). 2D Mater..

[cit107] Elibol K., Mangler C., ORegan D. D., Mustonen K., Eder D., Meyer J. C., Kotakoski J., Hobbs R. G., Susi T., Bayer B. C. (2021). ACS Nano.

[cit108] Meyer J. C., Kisielowski C., Erni R., Rossell M. D., Crommie M. F., Zettl A. (2008). Nano Lett..

[cit109] Meyer J. C., Girit C. O., Crommie M. F., Zettl A. (2008). Nature.

[cit110] Egerton R. (2013). Microsc. Microanal..

[cit111] Wehling T. O., Katsnelson M. I., Lichtenstein A. I. (2009). Phys. Rev. B: Condens. Matter Mater. Phys..

[cit112] Postl A., Hilgert P. P. P., Markevich A., Madsen J., Mustonen K., Kotakoski J., Susi T. (2022). Carbon.

[cit113] Tararan A., Zobelli A., Benito A. M., Maser W. K., Stéphan O. (2016). Chem. Mater..

[cit114] Chamberlain T. W., Biskupek J., Skowron S. T., Bayliss P. A., Bichoutskaia E., Kaiser U., Khlobystov A. N. (2015). Small.

[cit115] Erni R., Rossell M. D., Kisielowski C., Dahmen U. (2009). Phys. Rev. Lett..

[cit116] Mustonen K., Markevich A., Tripathi M., Inani H., Ding E.-X., Hussain A., Mangler C., Kauppinen E. I., Kotakoski J., Susi T. (2019). Adv. Funct. Mater..

[cit117] Markevich A., Hudak B. M., Madsen J., Song J., Snijders P. C., Lupini A. R., Susi T. (2021). J. Phys. Chem. C.

[cit118] Chang S. (2014). Phys. Today.

[cit119] Susi T. (2015). Res. Ideas Outcomes.

[cit120] Pool R. , Microscopy and Analysis, 2018

[cit121] Tripathi M., Mittelberger A., Pike N. A., Mangler C., Meyer J. C., Verstraete M. J., Kotakoski J., Susi T. (2018). Nano Lett..

[cit122] Nosraty Alamdary D., Kotakoski J., Susi T. (2017). Phys. Status Solidi B.

[cit123] Lee J., Zhou W., Pennycook S. J., Idrobo J.-C., Pantelides S. T. (2013). Nature.

[cit124] Yang Z., Yin L., Lee J., Ren W., Cheng H.-M., Ye H., Pantelides S. T., Pennycook S. J., Chisholm M. F. (2014). Angew. Chem., Int. Ed..

[cit125] Chen Q., Koh A. L., Robertson A. W., He K., Lee S., Yoon E., Lee G.-D., Sinclair R., Warner J. H. (2015). ACS Nano.

[cit126] Susi T., Skákalová V., Mittelberger A., Kotrusz P., Hulman M., Pennycook T. J., Mangler C., Kotakoski J., Meyer J. C. (2017). Sci. Rep..

[cit127] Dyck O., Kim S., Jimenez-Izal E., Alexandrova A. N., Kalinin S. V., Jesse S. (2018). Small.

[cit128] Markevich A. V., Baldoni M., Warner J. H., Kirkland A. I., Besley E. (2016). J. Phys. Chem. C.

[cit129] Susi T., Hofer C., Argentero G., Leuthner G. T., Pennycook T. J., Mangler C., Meyer J. C., Kotakoski J. (2016). Nat. Commun..

[cit130] Susi T., Meyer J. C., Kotakoski J. (2019). Nat. Rev. Phys..

[cit131] Yang S.-Z., Sun W., Zhang Y.-Y., Gong Y., Oxley M. P., Lupini A. R., Ajayan P. M., Chisholm M. F., Pantelides S. T., Zhou W. (2019). Phys. Rev. Lett..

[cit132] Zhao B., Shen D., Zhang Z., Lu P., Hossain M., Li J., Li B., Duan X. (2021). Adv. Funct. Mater..

[cit133] Ma Y., Li B., Yang S. (2018). Mater. Chem. Front..

[cit134] Susi T., Meyer J., Kotakoski J. (2017). Ultramicroscopy.

[cit135] Chirita A., Markevich A., Tripathi M., Pike N. A., Verstraete M. J., Kotakoski J., Susi T. (2022). Phys. Rev. B.

[cit136] Jesse S., Hudak B. M., Zarkadoula E., Song J., Maksov A., Fuentes-Cabrera M., Ganesh P., Kravchenko I., Snijders P. C., Lupini A. R., Borisevich A. Y., Kalinin S. V. (2018). Nanotechnology.

[cit137] Hudak B. M., Song J., Sims H., Troparevsky M. C., Humble T. S., Pantelides S. T., Snijders P. C., Lupini A. R. (2018). ACS Nano.

[cit138] Xu T., Shen Y., Yin K., Sun L. (2019). APL Mater..

[cit139] Dyck O., Ziatdinov M., Lingerfelt D. B., Unocic R. R., Hudak B. M., Lupini A. R., Jesse S., Kalinin S. V. (2019). Nat. Rev. Mater..

[cit140] Zhao X., Loh K. P., Pennycook S. J. (2021). J. Phys.: Condens. Matter.

[cit141] Kotakoski J., Meyer J. C., Kurasch S., Santos-Cottin D., Kaiser U., Krasheninnikov A. V. (2011). Phys. Rev. B: Condens. Matter Mater. Phys..

[cit142] Kretschmer S., Lehnert T., Kaiser U., Krasheninnikov A. V. (2020). Nano Lett..

[cit143] Lingerfelt D. B., Yu T., Yoshimura A., Ganesh P., Jakowski J., Sumpter B. G. (2021). Nano Lett..

[cit144] Yoshimura A., Lamparski M., Giedt J., Lingerfelt D., Jakowski J., Ganesh P., Yu T., Sumpter B. G., Meunier V. (2022). Nanoscale.

[cit145] Mangler C., Meyer J., Mittelberger A., Mustonen K., Susi T., Kotakoski J. (2022). Microsc. Microanal..

[cit146] Spurgeon S. R., Ophus C., Jones L., Petford-Long A., Kalinin S. V., Olszta M. J., Dunin-Borkowski R. E., Salmon N., Hattar K., Yang W.-C. D., Sharma R., Du Y., Chiaramonti A., Zheng H., Buck E. C., Kovarik L., Penn R. L., Li D., Zhang X., Murayama M., Taheri M. L. (2021). Nat. Mater..

[cit147] Ziatdinov M., Dyck O., Maksov A., Li X., Sang X., Xiao K., Unocic R. R., Vasudevan R., Jesse S., Kalinin S. V. (2017). ACS Nano.

[cit148] Madsen J., Postl A., Susi T. (2019). Microsc. Microanal..

[cit149] Postl A. , Nion Swift Atom Manipulator, 2022, https://github.com/arpostl/nionswift_atom_manipulator/

[cit150] Roccapriore K. M., Boebinger M. G., Dyck O., Ghosh A., Unocic R. R., Kalinin S. V., Ziatdinov M. (2022). ACS Nano.

[cit151] Dyck O., Jesse S., Kalinin S. V. (2019). MRS Bull..

